# Exploring Health Information-Seeking Preferences of Older Adults With Hypertension: Quasi-Experimental Design

**DOI:** 10.2196/cardio.8903

**Published:** 2018-05-30

**Authors:** Gabriele Sak, Peter Johannes Schulz

**Affiliations:** ^1^ Institute of Communication & Health Faculty of Communication Sciences Università della Svizzera italiana Lugano Switzerland

**Keywords:** desire for health information, assisted computer-based information search, decision self-efficacy, medical decision making, senior hypertensive patients, quasi-experimental design, Switzerland

## Abstract

**Background:**

Patients’ engagement in health care decision making is constituted by at least two behaviors: health information seeking and active involvement in medical decisions. Previous research reported that older adults desire a lot of information, but want to participate in decision making to a lesser degree. However, there is only limited evidence on the effect of desire for health information on seniors’ perceived confidence in making an informed choice (ie, decision self-efficacy).

**Objective:**

The goal of this study was to investigate the role desire for health information has for older patients. More specifically, it tested whether decision self-efficacy increases as a function of an assisted computer-based information search. Additionally, the study allowed insights into the sources seniors with hypertension prefer to consult.

**Methods:**

A sample of 101 senior citizens (aged ≥60 years) with high blood pressure in the Italian-speaking part of Switzerland answered a questionnaire before and after an informational intervention was applied. The intervention consisted of offering additional information on hypertension from five different sources and of providing the information the participant desired. Preference for receiving this information was the major independent variable. The main outcome measure was decision self-efficacy (assessed at baseline and posttest). Analyses of covariance were conducted to detect differences between and within who desired additional hypertension-related content (intervention group) and “information avoiders” (control group).

**Results:**

Health care professionals firmly remain the preferred and most trusted source of health information for senior patients. The second most consulted source was the internet (intervention group only). However, among the total sample, the internet obtained the lowest credibility score. A significant increase in decision self-efficacy occurred in seniors consulting additional information compared to information avoiders (F1,93=28.25, *P*<.001).

**Conclusions:**

Consulting health information on a computer screen, and assistance by a computer-savvy person, may be a helpful activity to increase perceived confidence in making treatment decisions in seniors with hypertension.

## Introduction

In health care, active involvement of patients has proved to be a beneficial but complex process to achieve, especially for the senior population. The active involvement of patients in their health care can manifest in at least two ways [1]. First, patients can actively request information from different sources and concerning different aspects of their treatment. Second, the patient can be involved in the medical decision-making process itself. Therefore, “desire for information and a wish to assume responsibility for decision making” are two distinct concepts in the context of patient participation (p 1144 [2]) [3]. Some measures of patients’ participation preferences are based exactly on this distinction, for example, the Krantz Health Opinion Survey [4]. The inconsistency between information seeking and willingness to contribute to medical decision making has also been reported in qualitative research with seniors [5].

There are a variety of different channels and sources that patients can use to consult information. The most important source may be the patient’s doctor [6-8]. “However, clinicians tend to underestimate the amount of information that patients require” [9-13]. Therefore, patients need to actively seek the information from their health care provider or other sources. This may be particularly challenging when older adults feel more passive and reliant on health care professionals [3,14]. Indeed, during consultations, older adults rarely ask important questions unless they are invited to [15]. When asked for the reason, many patients state that they “assume that the doctor would have told them everything relevant, others worry that they will appear foolish if they reveal their ignorance by asking questions, and some feel that they have already taken up too much of the busy doctor’s time” [13,15,16].

Being informed is a prerequisite for involvement in decision making and has been associated with outcomes including perceptions of quality of care, quality of life, psychological well-being, and improved health [17], as well as “adaptation to illness and treatment” [13,18,19], perception of control [20], and adherence [7,21].

Recent studies have investigated the antecedents to engage in a health information seeking. Apart from sociodemographic factors (eg, age, education), knowledge scores (eg, health and new media literacy), and disease complexity, information seeking was also influenced “by the individual’s personality characteristics, such as locus of control, self-efficacy, and preference for information” [19,22-24]. A recent study revealed that psychological empowerment proved to be a strong predictor of Swiss seniors’ ideal and actual role in treatment decisions [25].

To our knowledge, no study in Switzerland has yet examined the potential relationships between health information preference and decision self-efficacy with a sample composed of chronically ill seniors. To provide health-related information that was relevant to all potential participants, this study was condition-specific, with older adults (aged 60 years and older) suffering from high blood pressure. This condition was chosen because it requires constant treatment and thereby increases medical decision making (eg, [5,26]), and because one adult in four suffers from it in Switzerland [27].

This study tested whether decision self-efficacy increases as a function of desire for health information. Additionally, the experiment allowed insights into the antecedents of desire for information as well as the channels older patients prefer to consult.

In light of this, our first hypothesis is that participants showing higher desire for health information will report higher decision self-efficacy levels (measured at baseline and posttest) than seniors who did not engage in the consultation task offered (information avoiders). Moreover, we predict the desire to consult additional health information will be influenced by hypertension-related knowledge (hypothesis 2), psychological empowerment (hypothesis 3), eHealth literacy (hypothesis 4), trust in physician (hypothesis 5), and age (hypothesis 6).

## Methods

### Sampling Procedure

For this experiment, a sample composed of senior patients with hypertension (HT) was approached, between June and July 2017, via the following recruitment strategies: (1) older adults’ recreational or therapeutic day centers, (2) word of mouth, and (3) public settings such as bars and city parks. Seniors eligible for the study had to (1) be 60 years or older, (2) be residents in the Swiss-Italian region (Canton Ticino), (3) understand the Italian language, and (4) be formerly diagnosed with HT. To know the prospective participants’ HT status, the principal researcher had to ask if he/she was either currently suffering from high blood pressure or presently taking an antihypertensive drug. For respondents attending a recreational or therapeutic day center, the centers’ coordinators (or their assistants) indicated eligible seniors. A total of 101 senior patients with HT constituted the final sample. For time constraints, during the data collection process, HT status was assessed through a self-reported measure (ie, Charlson Comorbidity Index [CCI]) [28]. The study protocol was approved by the Ethical Review Board of the Università della Svizzera italiana on March 13, 2017.

### Intervention Procedure and Design

Before the study, all participants received relevant information about the researcher, his institution, and the aims of the study. After explicitly agreeing to participate in the study, respondents had to sign a written consent form. All respondents had to sign a form demonstrating their consent to participate in the controlled trial experiment.

An interview session consisted of a baseline measurement, the application of an assisted computer-based information search intervention, and a posttest measurement. Measurement was done by a self-administered computer questionnaire or face-to-face, depending on the respondent’s preference.

The intervention began with all participants reading a short text with basic information on HT shown on the computer screen. They then had to indicate whether they wished to obtain more information or not. If the participant desired further information, he or she was asked which from five sources they would like to consult: a doctor, a brochure, another HT patient, the Internet, or a friend (information seekers). If the participant did not desire additional information, he or she was asked to indicate the reasons why (information avoiders). For completion of the intervention, participants were given all the information pieces they desired. Information was provided as text on the computer screen or as a video. The information presented by each of the sources was edited according to the requirements of the source chosen. Each information source treated a different aspect related to this chronic condition or its management. Table 1 shows the five different health information sources with their respective topic designation and format (Multimedia Appendix 1 displays three information resources developed). The intervention was completed when the respondent had read all the information resources available or when the respondent felt he or she had read enough. The postintervention measurement was applied immediately after.

The basic information as well as the additional information were grounded on evidence-based information retrieved mainly from a brochure (“Arterial Hypertension: Information for Patients”) published by the Swiss Cardiology Association (*Fondazione Svizzera di Cardiologia*).

Classification of respondents into control group (ie, information avoiders) and experimental condition (ie, information seekers) was automatically achieved by the participant’s willingness to consult further information. In this sense, no randomization procedure could be applied. Therefore, this study represents a quasi-experimental design.

### Measurements

#### Baseline Survey Items/Scales

Subjective health status was measured through the single item developed by Renner and Schwarzer [29]. The presence of one or more chronic diseases was assessed via the CCI [28].

A set of single-items related to the participants’ HT condition (ad hoc created):

1. Hypertension degree asked seniors if they knew the degree (or severity) of their HT condition before adhering to the treatment regimen prescribed with response options: (1) first-degree HT, (2) second-degree HT, (3) third-degree HT, (4) systolic isolated HT, and (5) I don’t know/remember.
2. Hypertension personal history asked participants to report when they were diagnosed with HT.
3. Hypertension family history solicited participants to report whether any family member or relative suffered from HT.
4. Anti-HT drugs (names) asked whether the participant was currently taking one or more anti-HT drugs (dichotomous response option: no/yes). In case the participant answered affirmatively, he or she was prompted to recall the name(s) of each anti-HT drug.
5. Regular measurement of blood pressure asked HT patients if they are measuring their blood pressure levels on a regular basis (dichotomous response option: no/yes).
6. Smoke status simply asked respondents if they smoked on a regular basis.
7. Physical activity status asked seniors if they were currently participating in physical activity on a regular basis (walking on a regular basis was considered a physical activity).

A set of items related to health information and health information-seeking behavior:

1. General health information-seeking behavior (adapted from [30]) was measured via a single item asking: “Have you ever looked (or asked) for health-related information/advice (apart from that obtained from your doctor)?” (4-point scale ranging from 1=never to 4=very often).
2. Trust in different health information sources (adapted from [31]) was measured with the following item: “In general, how much would you trust information about health or medical topics obtained from each of the following sources?” (4-point scale ranging from 1=no trust at all to 4=a lot of trust).

Perceived competence in using information communication technologies (ICTs) was measured through the following single item: “How would you describe your competences in using ICTs?” (7-point scale ranging from 1=very bad to 7=very good).

The frequency of ICT use was collected via the following three single-items: “How often do you use the computer/smartphone/tablet?” (5-point scale ranging from 1=never to 5=always).

Internet health literacy (alpha=.96) was measured with the eHealth Literacy Scale (eHEALS) translated and validated into Italian by De Caro and colleagues (I-eHEALS [32]; original English version [33]).

Hypertension knowledge (alpha=.75) was evaluated with the Hypertension Knowledge-Level Scale (HK-LS) developed by Erkoc and colleagues [34]. In order to reduce the cognitive effort of the senior respondents in this study, only 11 (of 22) items yielding the higher factor loadings were retained (ie, two items comprising the “definition” subdimension and the first three items of the “medical treatment,” “drug compliance,” and “complications” subdimensions). To obtain the maximum score of 11, participants have to interpret four statements as “incorrect” and seven as “correct.” Incorrect statements were reverse coded. Higher values indicate higher hypertension knowledge.

Psychological empowerment (alpha=.94) was appraised with the multidimensional Spreitzer’s scale [35], but adapted to the HT context [36].

#### Independent Variable

The independent variable (intervention factor) was represented by the senior’s willingness to consult further information about hypertension (information seekers). Participants were asked the following two single-items: “Do you want to consult (or obtain) further information on the relevant aspects related to hypertension?” (ie, dichotomous response option: no/yes). The same question was asked for a maximum of five times. If the participant answered positively, he or she was asked: “From which of the following sources would you like to obtain further hypertension-related information?”

**Table 1:** Health information sources, topic designation, and formats.

Source	Content designation	Format
No source mentioned (basic)	Introduction on hypertension	Written on personal computer (PC)
Doctor^a^	The dangers of hypertension	Video on PC
Brochure^a^	The causes of hypertension	Screenshot of a brochure on PC
Other hypertension patient	Lifestyle change	Written on a screen
Internet^a^	Antihypertensive drugs	Screenshot of a webpage on PC
Friend	Blood pressure measurements	Written on PC

^a^Available in Multimedia Appendix 1.

#### Dependent Variable

The dependent variable was the Decision Self-Efficacy scale developed by O’Connor [37] (pretest: alpha=.96; posttest: alpha=.97). The scale (adapted to the HT context) “measures self-confidence or belief in one’s abilities in decision making, including shared decision making” (p 1 [37]). The measure is composed of 11 items (eg, “I feel confident that I can: get the facts about the anti-HT medication choices available to me” item 1) with five response categories ranging from 0=not at all confident to 4=very confident. All items are then summed, divided by 11, and multiplied by 25. Scores range from 0=very low self-efficacy to 100=very high self-efficacy (page 4 [37]).

#### Posttest Survey Items/Scales

Participant’s mood was evaluated through the Global Mood Scale developed by Denollet (positive affect: alpha=.86; negative affect: alpha=.94) [38].

Trust in physician (alpha=.88) was measured through the Abbreviated Wake Forest Physician Trust Scale (A-WFPTS) developed by Dugan and associates [39].

The posttest also included a set of sociodemographic items, such as gender, age, background origin, living situation, marital status, education, and length of doctor-patient relationship.

### Statistical Analyses

The collected data were analyzed quantitatively with SPSS version 21. Internal consistency tests were conducted to establish the reliability and validity of the main scales. Descriptive frequencies were provided for the main measures appraised. To assess the pure effect of the model conceptualized, and to partial out any potential variance of the groups at baseline, various independent-sample *t* tests and contingency coefficients were conducted. Different ANCOVAs were conducted to establish whether there were significant mean differences between the control group and the quasi-experimental condition while controlling for the influence of the outcome measure assessed at baseline. Factors assumed to be relevant for the variance of the outcome measure (ie, bivariate correlations), were included into the model as covariates. To provide more advanced analyses, the independent variable was dummy coded as follows: 0=no health information sources consulted, 1=one health information source consulted, and 2=two or more health information sources consulted. By doing so, planned contrasts can be derived from the ANCOVA tests. A priori power analysis was performed to provide an estimate of the acceptable sample size threshold. According to data from a pilot test (N=20), and in line with a recent study comparing older users and nonusers of online health information (N=225) [8], we set the control group average decision self-efficacy score to mean 48.3 (SD 23.6). As we hypothesized in our first hypothesis, we expected from the experimental group a 15-point increase in the average decision self-efficacy score (ie, mean 63.35). With an alpha=.05 and power beta=.90, the estimated sample needed with this effect size was approximately N=104.

## Results

### Participant Characteristics

A total of 107 participants showed initial interest to participate in the study. Of these, two did not complete the online survey and one, after a preliminary screening of the data, had his information discarded because he filled out the questionnaire inappropriately. Data from three additional seniors, who auto-completed the survey, were also discarded because they declared not to suffer from hypertension.

The final sample (N=101) was majority female, married or widowed seniors, with children, living independently at home, and with a Swiss-Italian or Italian background origin. All participants’ sociodemographic features can be seen in Table 2. Most respondents preferred to complete the survey as a face-to-face interview (n=97, 96.0%), rather than auto-complete it. Average duration time to complete the online survey was mean 35.11 (SD 11.47, range 14.88-67.12) minutes.

### Main Analyses

#### Control and Quasi-Experimental Group

Of 101 senior respondents, 60 (59.4%) decided not to consult further health information (information avoider), whereas the remaining 41 showed interest in reading more information related to HT (40.6%; information seekers). The majority of these 41 individuals decided to stop the guided information search task after one health information resource (70%), 10 accessed two information resources (24%), and only two participants consulted three information sources In the first consulting session, 15 participants preferred to watch the doctor’s piece of information (ie, video format; 37%), eight the Internet webpage (20%), and six seniors selecting either a brochure’s screenshot (15%), a text written by another HT patient (15%) or contents disclosed by a friend (15%). None of the participants consulted more than three different sources out of a maximum of five. These findings confirm the dominant position of health care providers and the emergent interest in turning to the Internet to obtain health information. In light of the small sample size, all main analyses were conducted for the two main groups, namely “health information avoiders” and those health information seekers.

**Table 2:** Participants’ characteristics for the total sample and by intervention groups.

Item/Scale		Total (N=101)	Health information seekers (n=41)	Health information avoiders (n=60)
Gender (male), n (%)		38 (37.6)	11 (26.8)	27 (45)
Age (years), mean (SD)		74.9 (8.1)	74.4 (7.1)	75.3 (8.7)
**Marital status, n (%)**				
	Married	50 (49.5)	22 (53.7)	28 (46.7)
	Widowed	34 (33.7)	15 (36.6)	19 (31.7)
	Separated	4 (4.0)	—^a^	4 (6.7)
	Divorced	8 (7.9)	4 (9.8)	4 (6.7)
	Single (never married)	5 (5.0)	—	5 (8.3)
**Children (yes), n (%)**		90 (89.1)	37 (90.2)	53 (88.3)
	Number of children, mean (SD)	2.4 (1.1)	2.3 (1.1)	2.5 (1.2)
**Living situation, n (%)**				
	I live independently at home	71 (70.3)	31 (75.6)	40 (66.7)
	I live at home with the support of family members	12 (11.9)	5 (12.2)	7 (11.7)
	I live at home with the social service support (eg, food)	7 (6.9)	—	7 (11.7)
	I live at home but I receive a homecare service (eg, SCUDO)	8 (7.9)	4 (9.8)	4 (6.7)
	I live in a retirement house	3 (3.0)	1 (2.4)	2 (3.3)
Day center attendance (yes), n (%)		51 (50.5)	18 (43.9)	33 (55)
**Background origin, n (%)**				
	Swiss-German	7 (6.7)	2 (4.9)	5 (8.3)
	Swiss-Italian	49 (48.5)	18 (48.9)	31 (51.7)
	Italian (Italy)	25 (24.8)	13 (31.7)	12 (20.0)
	German (Germany)	1 (1.0)	1 (2.4)	—
	French (France)	1 (1.0)	—	1 (1.7)
	Other origins	18 (17.8)	7 (17.1)	11 (18.3)
**Educational level, n (%)**				
	No degree obtained	9 (8.7)	1 (2.4)	8 (13.3)
	Primary school degree	39 (38.6)	15 (36.6)	24 (40.0)
	Apprenticeship degree	22 (21.8)	10 (24.4)	12 (20.0)
	College or similar degree	19 (18.8)	11 (26.8)	8 (13.3)
	Applied university degree	1 (1.0)	—	1 (1.7)
	University of polytechnic degree	11 (10.9)	4 (9.8)	7 (11.7)
Health-related profession (yes), n (%)		13 (12.9)	5 (12.2)	8 (13.3)
Doctor-patient relationship length (years), mean (SD)		17.5 (11.8)	14.7 (11.4)	19.5 (11.7)^b^
Doctor visits a year, mean (SD)		3.6 (3.2)	3.5 (3.1)	3.6 (3.2)

^a^No participants apply to item.

^b^*P*=.047.

#### Between-Group Differences

No significant differences were found between information seekers and avoiders in terms of gender, age, marital status, living situation, having children, education, day center attendance, health status, comorbidity index, hypertension personal and family history, smoke status, physical activity status, perceived competence in using ICTs, eHealth literacy, positive and negative mood subscales, trust in physician, and number of visits with the doctor per year.

However, some significant between-group differences emerged: information seekers, in comparison with information avoiders, were more aware of their HT condition (73% vs 52%), conducted more blood pressure measurements on a regular basis (83% vs 60%), used a mobile phone more frequently (mean 3.12, SD 1.81 vs mean 2.38, SD 1.80), perceived the ability to access health-related resources online as more important (mean 3.12, SD 1.33 vs mean 2.48, SD 1.30), had higher trust in online health information sources (mean 1.61, SD 0.77 vs mean 1.32, SD 0.62) and magazines (mean 3.02, SD 1.04 vs mean 2.38, SD 1.04), and had a shorter average doctor-patient relationship length (mean 14.73, SD 11.44 vs mean 19.45, SD 11.69). At the same time, the quasi-experimental group reported higher decision self-efficacy (at baseline: mean 57.37, SD 20.19 vs mean 44.96, SD 26.98), psychological empowerment (mean 2.47, SD 0.67 vs mean 1.94, SD 0.75), and hypertension-related knowledge (mean 7.66, SD 1.94 vs mean 6.12, SD 2.80) levels than the information avoiders. To address the assumption regarding independence of the covariate and treatment effect [40], all variables achieving a significant difference between the two subsets of the sample were not included into subsequent analyses. The only exception was decision self-efficacy, which was the main outcome measure and the only scale administered both at baseline and posttest.

#### Hypothesis Testing

To identify all the relevant covariates that might influence the posttest scores of decision self-efficacy, bivariate correlation coefficients were derived. Pearson correlation coefficients equal to or greater than *r=*.40 (moderate effect size; [41]), were included in the analysis of covariance. Furthermore, to be included into the main analyses, a covariate had to satisfy the assumption of independence of the treatment effect. Table 3 shows the bivariate correlations obtained and the presence or absence of any between-group differences on the two groups of the study.

**Table 3:** Bivariate correlations between decision self-efficacy (posttest) and its antecedents, including between-group differences.

Item/scale^a^		Correlation of decision self-efficacy (posttest) and its antecedents, *r*	Between-group differences^b^
1	DSE Scale (baseline)	.94^c^	Yes
2	HK-LS	.55^c^	Yes
3	Health Empowerment Scale	.71^c^	Yes
4	ICTs’ Perceived Competence	.59^c^	No
5	I-eHEALS	.67^c^	No
6	General (HIS) behavior	.63^c^	Yes
7	Positive mood subscale (GMS)	.41^c^	No
8	Negative mood subscale (GMS)	–.41^c^	No
9	Education	.59^c^	No
10	Age	–.24^d^	No
11	Health status	.28^c^	No
12	Comorbidity index (CCI)	–.25^d^	No
13	Doctor visits (per year)	–.28^c^	No
14	Doctor-patient relationship length	–.24^d^	Yes
15	Trust-in-physician scale (A-WFPTS)	–.34^c^	No
16	HT personal history (years with HT)	–.15	No

^a^A-WFPTS: Abbreviated Wake Forest Physician Trust Scale; CCI: Charlson Comorbidity Index; DSE: Decision Self-Efficacy; GMS: Global Mood Scale; HIS: health information seeker; HK-LS: Hypertension Knowledge-Level Scale; HT: hypertension; ICT: information communication technology; I-eHEALS: Italian version of the eHealth Literacy Scale.

^b^Independent-sample *t* tests between control and intervention group.

^c^*P*<.001.

^d^*P*<.05.

Based on the two previous tests, main variables included into the model as covariates were decision self-efficacy (baseline), eHealth literacy, positive and negative affect subscales of the GMS, education, and perceived competence in using ICTs.

Our first hypothesis forecast that decision self-efficacy of seniors with HT increases as a function of the preference for more health information. The main covariates included in the model were decision self-efficacy assessed at baseline and eHealth literacy, whereas the independent variable was the willingness to obtain supplementary health information. The ANCOVA test revealed that decision self-efficacy assessed at baseline was significantly related to seniors’ decision self-efficacy evaluated in the posttest (*F*_1,93_=307; *P*<.001, *r*=.88). By interpreting the estimates of effect size derived, we can conclude that baseline levels of decision self-efficacy had a very strong influence on the outcome measure’s scores (*r*=.88). All other covariates yielded nonsignificant relationships with the dependent variable. On the other hand, there was a statistically significant main effect of the treatment condition on levels of decision self-efficacy measured at posttest while controlling for the effects of decision self-efficacy levels in the preexperimental phase, eHealth literacy, and the remaining covariates of the model (*F*_1,93_=28.25, *P*<.001; partial η^2^=.23). Based on these findings, we can accept our first hypothesis. Analyses were repeated with the independent variable dummy coded into three groups: 0=no health information sources consulted (n=60), 1=one health information source consulted (n=29), and 2=two or more health information sources consulted (n=12). The same variables confirmed the findings. Planned contrasts revealed that both treatment conditions increased posttest decision self-efficacy compared to the control group (one health information source consulted: *t*_92_=8.48, *P*<.001; two or more health information sources consulted: *t*_92_=10.73, *P*<.001). Nevertheless, pairwise comparisons showed that consulting two or more health information sources rather than only one did not significantly increase posttest decision self-efficacy levels.

All the remaining hypotheses could be tested by the same independent-sample *t* tests conducted to spot any between-group difference (refer to Between-Group Differences). Health information seekers reported significantly higher scores on the hypertension-related knowledge and psychological empowerment scales than information avoiders; therefore, our hypotheses that the desire to consult additional health information will be influenced by hypertension-related knowledge (hypothesis 2) and psychological empowerment (hypothesis 3) were confirmed. On the other hand, differences were not detected in levels of eHealth literacy, trust in physician, and age; therefore, our hypotheses that the desire to consult additional health information will be influenced by eHealth literacy (hypothesis 4), trust in physician (hypothesis 5), and age (hypothesis 6) were all rejected.

## Discussion

### Principal Results and Prior Work

Overall, the present quasi-experiment showed that seniors with HT wishing to engage in an assisted consulting health information session via a personal computer substantially increased their perceived confidence in making an informed choice about treatment (ie, decision self-efficacy). This finding is in line with past evidence, which established a relationship between fulfillment of health information needs and perceptions of control [19,20,22,23,42]. As the two quasi-experimental groups did not differ in terms of posttest decision self-efficacy average scores, it may be assumed that the number of health resources consulted is not as influential as the mere willingness to obtain supplementary contents in general. Nevertheless, due to the small and unequal split of the sample, this advanced analysis (ie, pairwise comparisons) has to be considered with caution and possibly tested in future studies reaching bigger samples. The impact of receiving the health information desired is not only ascribed to foster seniors’ self-efficacy and empowerment beliefs. Indeed, past evidence has also demonstrated that the influence of such a construct has to be extended also to actual behavior change and health outcomes, such as improved adherence in general [21,43], and for senior patients with HT in particular [7]. This indicates the pronounced influence of fulfilling health information needs on psychological and heath-enhancing behaviors.

Moreover, this study aimed to investigate the antecedents of seniors’ health information needs. As expected, knowledge about hypertension, psychological empowerment, and decision self-efficacy (at baseline) largely contributed to distinguish senior information seekers from the so-called “information avoiders.” These results are also consistent with available evidence investigating health information-seeking behaviors [19,22-24,42]. Based on our results, adult patients with HT desiring more information feel more empowered and self-confident in making medical decisions compared to avoiders and are more knowledgeable about their HT condition, conduct more blood pressure measurements on a regular basis, and attribute higher trust/importance on online health information resources.

It has to be noted that levels of decision self-efficacy (posttest) were highly correlated with psychological empowerment, hypertension-related knowledge, education, eHealth literacy, positive and negative mood (negative correlation), perceived competence in using ICTs, and past information-seeking behavior. In this sense, their large influence on seniors’ perceived confidence in making informed decisions about anti-HT treatment have to investigated further. Future experimental efforts have to be designed with more sophisticated randomization procedures to ultimately allow the inclusion of all these relevant constructs.

In terms of trust toward different health-related resources, health care professionals remain the most trusted source for senior patients with HT, and the Internet is the least trusted. Seniors not engaging in the assisted health information search (information avoiders) rated as the second most trusted source family members and/or friends, whereas information seekers ranked magazines as the second most reliable channel. This divergence might be explained by the fact that health information avoiders prefer to approach a “living source” of information, which requires less effort and skills to access it [44]. The majority of information seekers preferred to obtain contents disseminated by a health care provider followed by the Internet. These results are consistent with previous research emphasizing the prominent role of doctors as preferred health information source [6,8,45] and the emergent interest of seniors toward online health resources [8,46-48]. Nevertheless, to properly use this technology to access health content*-, and thereby satisfy this “e-interest,” seniors need specific guidance and training (ie, computer and eHealth literacy; [49]). Indeed, according to Chew and Yuqian, “eHealth literacy empowers individuals to take better care of their health and can be enhanced through training” (p 323 [50]). In turn, this might lower the high level of mistrust that senior patients actually attribute to the Internet [51]. At the same time, future efforts developing measures to assess “Internet-based decision aid tools to determine how better to advise and direct patients to useful online decision tools” are warranted (p 757 [8]).

### Limitations

First, due to time constraints, the study did not use a random sample. Therefore, reported findings are generalizable only to adults (60 years and older) with hypertension, and residing in the Italian-speaking region of Switzerland. Second, the unequal split between the control and quasi-experimental conditions, coupled with the lack of a proper randomization technique, hindered the possibility to derive more sophisticated results. Indeed, main analyses were conducted only for the two main groups (information seekers vs avoiders), and the inclusion of control variables presumed to be relevant was limited (ie, knowledge about hypertension, empowerment). Third, because the majority of respondents preferred to complete the survey face-to-face (96%), and the principal researcher conducted most of them (92%), a potential interviewer effect could not be excluded. Moreover, participants may have answered the survey items in a socially desirable way in front of the interviewer. The application of self-reported measures is also subject to social desirability and response bias.

Finally, approximately 40% of the respondents were approached in public locations (eg, bars, parks). This aspect may have limited their willingness to initiate (or extend) the assisted health information task offered. Indeed, none of the respondents consulted more than three different sources out of a maximum of five.

### Conclusion

Our quasi-experiment investigated the effects of an assisted computer-based health information search on seniors’ perceived confidence in making informed decisions about HT treatments (decision self-efficacy). Antecedents of seniors’ desire for detailed health information, trust, and preferred sources of HT-related content were also evaluated. The results showed significant differences between senior information seekers and avoiders. Engaging in a guided computer-based health information search session proved to be a helpful approach boosting seniors’ perceived ability to make treatment decisions. Future research has to empirically replicate these findings with a representative random sample of seniors with HT. Qualitative research may also be helpful to explore in depth the findings of this study. Although health care professionals remain the dominant source of health information for older adults, new patterns of health information seeking emerged. The growing interest of the Internet as a health information channel might positively influence involvement preferences in medical decision making by satisfying information needs of chronically ill seniors. Nevertheless, to overcome the perceived obstacles to use the World Wide Web, and foster acceptance and trust among elderly people, educational interventions are required (eHealth literacy). When designing health education materials, public health organizations have to tailor health information (ie, with different formats and styles) in a way that will equally satisfy and reach seniors with high information needs and those showing a low desire for accessing health content.

## Appendix

Multimedia Appendix 1 Health information resources.

## Abbreviations

A-WFPTS: Abbreviated Wake Forest Physician Trust Scale
CCI: Charlson Comorbidity Index
DSE: Decision Self-Efficacy
eHEALS: eHealth Literacy Scale
GMS: Global Mood Scale
HIS: health information seeker
HK-LS: Hypertension Knowledge-Level Scale
HT: hypertension
ICT: information and communications technology
I-eHEALS: Italian eHealth Literacy Scale
