# Supplementary material for: Exploring Health Information-Seeking Preferences of Older Adults With Hypertension: Quasi-Experimental Design
Source: JMIR Cardio. 2018 May 30;2(1):e12. doi: 10.2196/cardio.8903 (PMC6834236; doi:10.2196/cardio.8903)
Supplement: Multimedia Appendix 1 [file cardio_v2i1e12_app1.pdf]

Multimedia Appendix 1

Figure 1. Health information resource - Brochure about the causes of hypertension

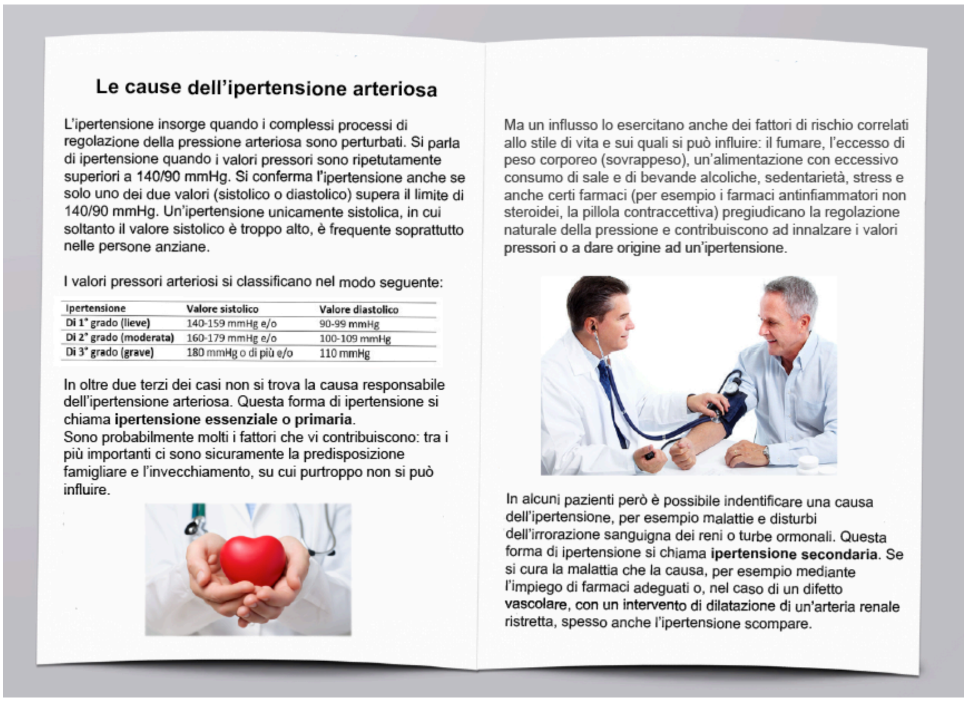

Le cause dell'ipertensione arteriosa

L'ipertensione insorge quando i complessi processi di regolazione della pressione arteriosa sono perturbati. Si parla di ipertensione quando i valori pressori sono ripetutamente superiori a 140/90 mmHg. Si conferma l'ipertensione anche se solo uno dei due valori (sistolico o diastolico) supera il limite di 140/90 mmHg. Un'ipertensione unicamente sistolica, in cui soltanto il valore sistolico è troppo alto, è frequente soprattutto nelle persone anziane.

I valori pressori arteriosi si classificano nel modo seguente:

Ipertensione	Valore sistolico	Valore diastolico
Di 1° grado (lieve)	140-159 mmHg e/o	90-99 mmHg
Di 2° grado (moderata)	160-179 mmHg e/o	100-109 mmHg
Di 3° grado (grave)	180 mmHg o di più e/o	110 mmHg

In oltre due terzi dei casi non si trova la causa responsabile dell'ipertensione arteriosa. Questa forma di ipertensione si chiama **ipertensione essenziale o primaria**.
Sono probabilmente molti i fattori che vi contribuiscono: tra i più importanti ci sono sicuramente la predisposizione famigliare e l'invecchiamento, su cui purtroppo non si può influire.

Ma un influsso lo esercitano anche dei fattori di rischio correlati allo stile di vita e sui quali si può influire: il fumare, l'eccesso di peso corporeo (sovrappeso), un'alimentazione con eccessivo consumo di sale e di bevande alcoliche, sedentarietà, stress e anche certi farmaci (per esempio i farmaci antinfiammatori non steroidei, la pillola contraccettiva) pregiudicano la regolazione naturale della pressione e contribuiscono ad innalzare i valori pressori o a dare origine ad un'ipertensione.

In alcuni pazienti però è possibile indentificare una causa dell'ipertensione, per esempio malattie e disturbi dell'irrorazione sanguigna dei reni o turbe ormonali. Questa forma di ipertensione si chiama **ipertensione secondaria**. Se si cura la malattia che la causa, per esempio mediante l'impiego di farmaci adeguati o, nel caso di un difetto vascolare, con un intervento di dilatazione di un'arteria renale ristretta, spesso anche l'ipertensione scompare.

Figure 2. Health information resource - Video about the dangers of hypertension

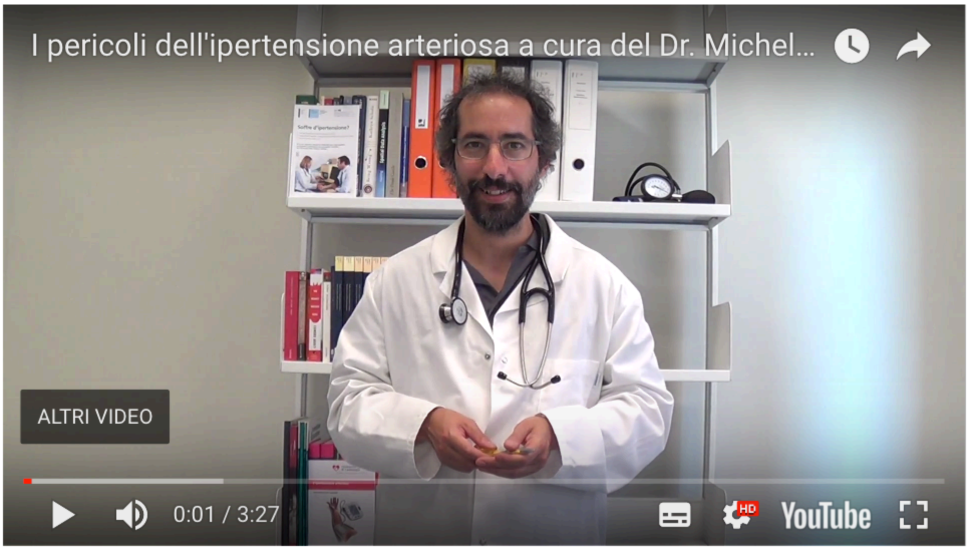

Figure 3. Health information resource - Webpage about anti-hypertensive drugs

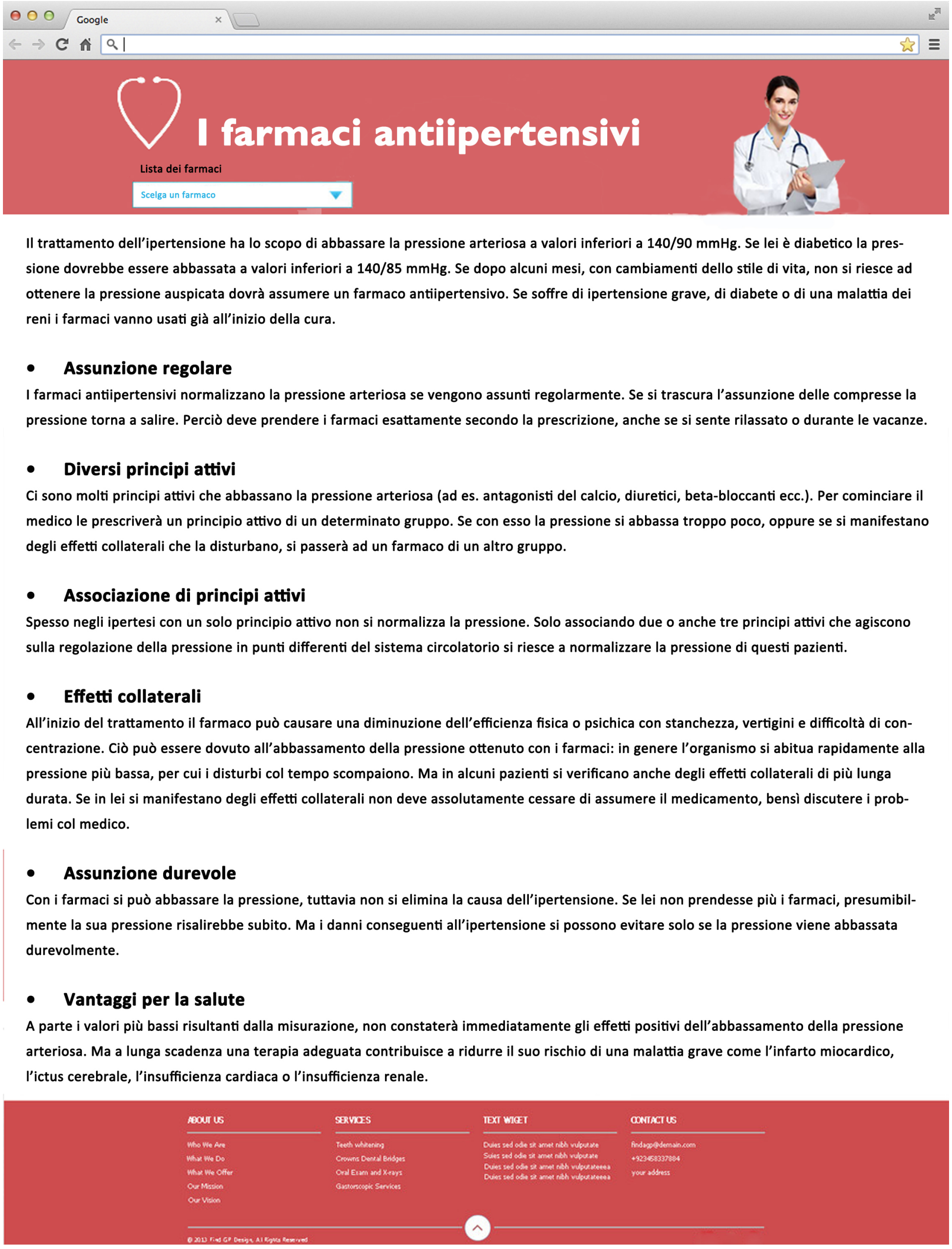

Google

I farmaci antiipertensivi

Lista dei farmaci

Scelga un farmaco

Il trattamento dell'ipertensione ha lo scopo di abbassare la pressione arteriosa a valori inferiori a 140/90 mmHg. Se lei è diabetico la pressione dovrebbe essere abbassata a valori inferiori a 140/85 mmHg. Se dopo alcuni mesi, con cambiamenti dello stile di vita, non si riesce ad ottenere la pressione auspicata dovrà assumere un farmaco antiipertensivo. Se soffre di ipertensione grave, di diabete o di una malattia dei reni i farmaci vanno usati già all'inizio della cura.

- **Assunzione regolare**

I farmaci antiipertensivi normalizzano la pressione arteriosa se vengono assunti regolarmente. Se si trascura l'assunzione delle compresse la pressione torna a salire. Perciò deve prendere i farmaci esattamente secondo la prescrizione, anche se si sente rilassato o durante le vacanze.

- **Diversi principi attivi**

Ci sono molti principi attivi che abbassano la pressione arteriosa (ad es. antagonisti del calcio, diuretici, beta-bloccanti ecc.). Per cominciare il medico le prescriverà un principio attivo di un determinato gruppo. Se con esso la pressione si abbassa troppo poco, oppure se si manifestano degli effetti collaterali che la disturbano, si passerà ad un farmaco di un altro gruppo.

- **Associazione di principi attivi**

Spesso negli ipertesi con un solo principio attivo non si normalizza la pressione. Solo associando due o anche tre principi attivi che agiscono sulla regolazione della pressione in punti differenti del sistema circolatorio si riesce a normalizzare la pressione di questi pazienti.

- **Effetti collaterali**

All'inizio del trattamento il farmaco può causare una diminuzione dell'efficienza fisica o psichica con stanchezza, vertigini e difficoltà di concentrazione. Ciò può essere dovuto all'abbassamento della pressione ottenuto con i farmaci: in genere l'organismo si abitua rapidamente alla pressione più bassa, per cui i disturbi col tempo scompaiono. Ma in alcuni pazienti si verificano anche degli effetti collaterali di più lunga durata. Se in lei si manifestano degli effetti collaterali non deve assolutamente cessare di assumere il medicamento, bensì discutere i problemi col medico.

- **Assunzione durevole**

Con i farmaci si può abbassare la pressione, tuttavia non si elimina la causa dell'ipertensione. Se lei non prendesse più i farmaci, presumibilmente la sua pressione risalirebbe subito. Ma i danni conseguenti all'ipertensione si possono evitare solo se la pressione viene abbassata durevolmente.

- **Vantaggi per la salute**

A parte i valori più bassi risultanti dalla misurazione, non constaterà immediatamente gli effetti positivi dell'abbassamento della pressione arteriosa. Ma a lunga scadenza una terapia adeguata contribuisce a ridurre il suo rischio di una malattia grave come l'infarto miocardico, l'ictus cerebrale, l'insufficienza cardiaca o l'insufficienza renale.

ABOUT US
Who We Are
What We Do
What We Offer
Our Mission
Our Vision

SERVICES
Teeth whitening
Crowns Dental Bridges
Oral Exam and X-rays
Gastorscopic Services

TEXT WIGET
Duies sed odie sit amet nibh vulputate
Suies sed odie sit amet nibh vulputate
Duies sed odie sit amet nibh vulputateeea
Duies sed odie sit amet nibh vulputateeea

CONTACT US
findagp@demain.com
+923458337884
your address

© 2013 Find GP Design, All Rights Reserved
